# Structural Basis for the Interaction between p53 Transactivation Domain and the Mediator Subunit MED25

**DOI:** 10.3390/molecules23102726

**Published:** 2018-10-22

**Authors:** Min-Sung Lee, Kyungeun Lim, Mi-Kyung Lee, Seung-Wook Chi

**Affiliations:** 1 Disease Target Structure Research Center, KRIBB, Daejeon 34141, Korea; mslee@kribb.re.kr (M.-S.L.); kel3833@gmail.com (K.L.); 2 Department of Proteome Structural Biology, KRIBB School of Bioscience, Korea University of Science and Technology, Daejeon 34113, Korea

**Keywords:** p53, MED25, transactivation domain, protein-protein interaction, complex structure, nuclear magnetic resonance

## Abstract

Eukaryotic transcription initiation is mediated by interactions between transcriptional activators and the mediator coactivator complex. Molecular interaction of p53 transcription factor with mediator complex subunit 25 (MED25) is essential for its target gene transcription. In this study, we characterized the molecular interaction between p53 transactivation domain (p53TAD) and activator interaction domain (ACID) of MED25 using nuclear magnetic resonance (NMR) spectroscopy. The NMR chemical shift perturbation and isothermal titration calorimetry (ITC) data showed that p53TAD interacted with MED25 ACID mainly through the p53TAD2 sequence motif. Taken together with the mutagenesis data, the refined structural model of MED25 ACID/p53TAD2 peptide complex showed that an amphipathic α-helix of p53TAD2 peptide bound an elongated hydrophobic groove of MED25 ACID. Furthermore, our results revealed the highly conserved mechanism of MED25 interaction with intrinsically unfolded acidic TADs from the transcriptional activators p53, ERM (Ets-related molecule), and herpes simplex virus protein 16 (VP16).

## 1. Introduction

**Figure 1:**
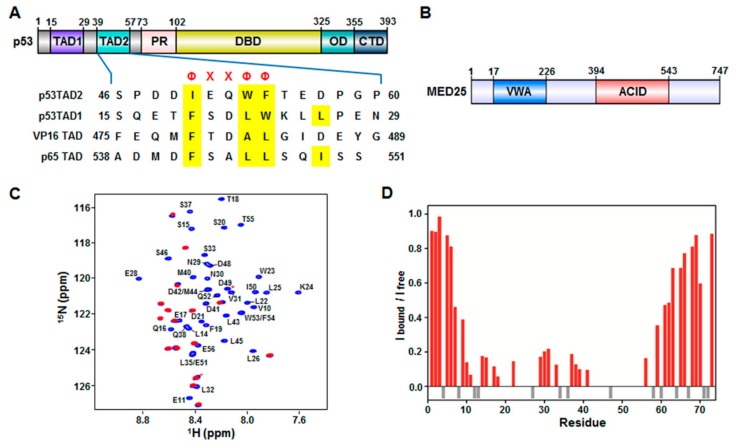
Interaction of mediator complex subunit (MED) 25 activator interaction domain (ACID) with p53TAD. (**A**) Domain organization of p53 and sequence alignment of p53TAD with acidic TADs in VP16 and p65. p53 consists of transactivation domain (TAD), proline-rich domain (PR), DNA-binding domain (DBD), oligomerization domain (OD), and C-terminal domain (CTD). Φ and Χ indicate a bulky hydrophobic residue and any other residue, respectively; (**B**) Domain organization of MED25. MED25 contains von Willebrand factor A (VWA) domain and activator interaction domain (ACID); (**C**) Overlay of 2D ^1^H-^15^N heteronuclear single-quantum correlation (HSQC) spectra of ^15^N-labeled p53TAD in the absence (blue) or presence (red) of MED25 ACID at a molar ratio of 1:2. The cross-peaks of ^15^N-labeled p53TAD alone (blue) are represented by residues types and sequence numbers; (**D**) Cross-peak intensity ratio (I_bound_/I_free_) for p53TAD (red). Gray negative bars indicate proline residues.

The tumor suppressor p53 is a transcription factor that plays essential roles in various cellular processes, including cell cycle arrest, DNA repair, and apoptosis in response to stress signals such as DNA damage, hypoxia, oncogene activation, and viral infection [1,2,3,4]. Within the nucleus, p53 mediates its cellular functions through transcriptional activation of target genes encoding pro-apoptotic proteins such as B-cell lymphoma 2-associated X protein (BAX) and p53 upregulated modulator of apoptosis (PUMA) and cyclin kinase inhibitors like p21. p53 contains distinct structural and functional domains: a N-terminal transactivation domain (TAD) (residues 1–73), a central DNA-binding domain (DBD) (residues 102–325), an oligomerization domain (OD) (residues 325–355), and a C-terminal regulatory domain (CTD) (residues 356–393) (Figure 1A). During transcription, p53 binds enhancer regions of target genes and assembles the transcriptional machinery such as RNA polymerase II (Pol II) general transcription factors (GTFs), and mediator, mainly through its DBD and TAD.

Within the transcriptional machinery, the mediator coactivator complex acts as a central integrator of transcription [5]. The mediator coactivator complex interacts with a variety of transcriptional activators and recruits RNA Pol II to induce target gene transcription. The mediator coactivator complex plays key roles in transmitting the signals from the transcription factors to the RNA Pol II through its association with them. The interaction between the mediator and transcription factors activates the transcription by inducing the formation of a large pocket domain at the mediator-RNA Pol II interaction site [6]. Previously, it has been reported that the mediator complex subunit 17 (MED17) [7] and MED1 [8] interact specifically with the transcription factor p53. MED25 was newly identified as a critical target protein of various transcriptional activators [9,10]. Previously, it has been shown that MED25 interacts with the viral transactivators herpes simplex virus protein 16 (VP16) [11,12], immediate early 62 protein (IE62) [13,14] and lana-1 [15], the endoplasmic reticulum stress-responsive transcription factor ATF6α [16], and the *Arabidopsis* transcriptional regulator DREB2A [17,18]. More recently, it has also been demonstrated that MED25 binds the human transcription factor ERM/ETV5, a PEA3 group member of the Ets family transcription factors [19,20].

MED25 is composed of two distinct functional domains, a von Willebrand factor A (VWA) domain (residues 17–226) that anchors to mediator and an activator interaction domain (ACID) (residues 394–543) that binds to transcription factors (Figure 1B). The VP16 transactivator interacts with MED25 ACID via its TAD [11,12]. The VP16 TAD (residues 412–490) and p53TAD (residues 1–73) are acidic and intrinsically unfolded [21,22]. They include two independent subdomains that are referred to as H1 (residues 410–452) and H2 (residues 453–490) in VP16, and p53TAD1 and p53TAD2 in p53 (Figure 1A). Both subdomains of VP16 and p53 have not only acidic amino acids but also key hydrophobic and aromatic amino acids that are involved in the interaction with transcriptional regulatory proteins to activate transcription [21,22]. Because of intrinsic disorder of acidic TADs, little is known about the structural basis of their interaction with various transcriptional activators.

In this study, we characterized the molecular interaction between p53TAD (1–73) (including TAD1 with residues 15–29 and TAD2 with residues 39–57) and MED25 ACID by nuclear magnetic resonance (NMR) spectroscopy. Taken together with the isothermal titration calorimetry (ITC) data, the NMR data showed that p53TAD directly interacted with MED25 ACID, and the p53TAD2 peptide (residues 39–57) is mainly responsible for interaction with MED25 ACID. The refined structural model revealed that the α-helix of p53TAD2 peptide fitted into a hydrophobic groove composed of the central β-barrel and α1-helix in MED25 ACID. Our results provided the structural basis for the conserved interaction of MED25 ACID with intrinsically unfolded acidic TADs of transcriptional activators.

## 2. Results and Discussion

### 2.1. Interaction between p53TAD and MED25 ACID

Previously, it was shown that MED25 ACID interacts with acidic TADs of transcriptional activators such as VP16 TAD [11,12] and ERM TAD [19,20]. Based on sequence similarity between VP16 TAD and p53TAD (Figure 1), we hypothesized that p53TAD was also able to interact with MED25 ACID. To test our hypothesis, we performed the binding titration experiments between full-length p53TAD (residues 1–73) and MED25 ACID using NMR spectroscopy. The addition of unlabeled MED25 ACID into ^15^N-labeled p53TAD caused severe line broadening in a majority of crosspeaks (Figure 1C), indicating that the binding involved the intermediate to slow exchange between free and MED25 ACID-bound forms on the NMR chemical shift time scale. Based on the previously reported assignments of free p53TAD [23], the backbone resonances for MED25 ACID-bound p53TAD were assigned, and the intensity ratio was plotted against each residue of p53TAD during the NMR titration (Figure 1D). The residues showing a significant decrease in intensity of crosspeaks upon binding to MED25 ACID were mainly located in the p53TAD1 region (Phe19-Leu25) as well as in the p53TAD2 region (Leu45-Thr55). As shown in Figure 1A, both p53TAD1 and p53TAD2 regions contained a ΦΧΧΦΦ (Φ: a bulky hydrophobic residue, X: any other residue) sequence motif, which acted as a binding site for other protein partners such as murine double minute 2 (MDM2) [24,25], transcriptional coactivator p300/CREB-binding protein (CBP) [26,27,28,29,30], mitochondrial transcription factor A [23], mitochondrial single-stranded DNA-binding protein [31], positive cofactor 4 [32], and breast cancer type 2 susceptibility protein [33]. Our results suggest that the interaction between p53TAD and MED25 ACID may involve both ΦΧΧΦΦ-binding motifs, p53TAD1 and p53TAD2.

### 2.2. Mapping of the p53TAD-Binding Surface on MED25 ACID

**Figure 2:**
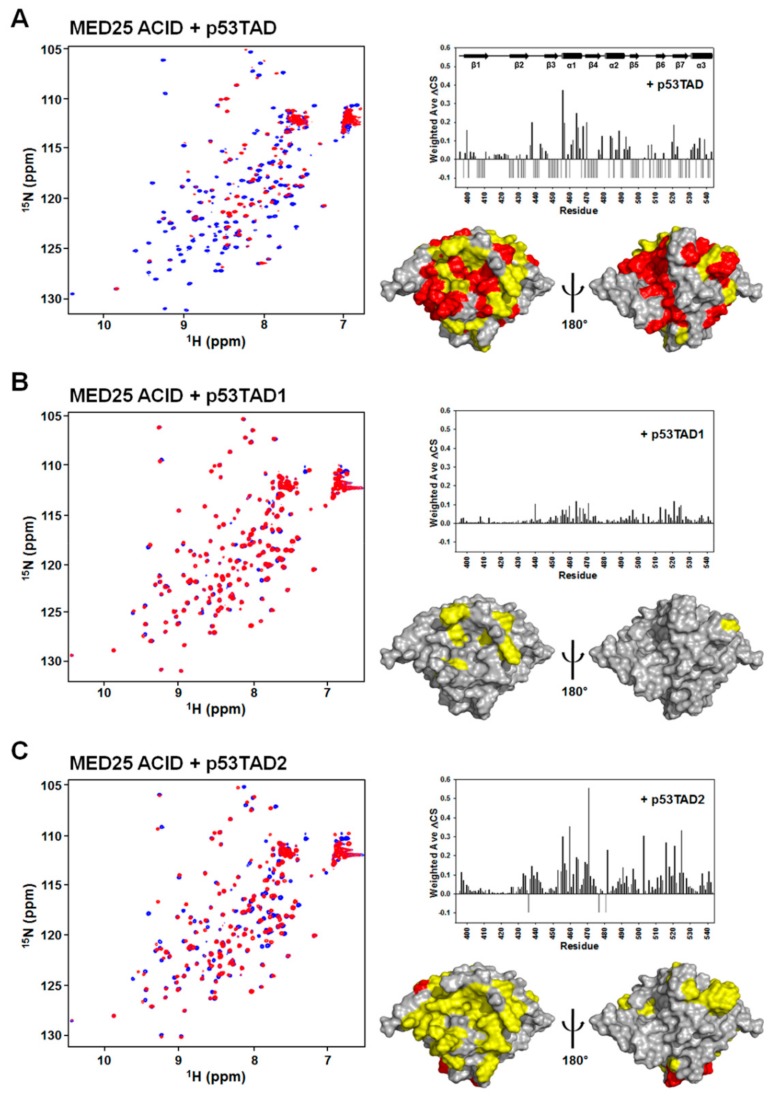
Mapping of p53TAD-binding surface of MED25 ACID by NMR. (**A**) Overlay of 2D ^1^H-^15^N HSQC spectra of ^15^N-labeled MED25 ACID in the absence (blue) or presence (red) of full-length p53TAD, p53TAD1, and p53TAD2 peptides at a molar ratio of 1:1; (**B**) Chemical shift perturbations on MED25 ACID induced by full-length p53TAD, p53TAD1, and p53TAD2 binding. Weighted ΔCS values were calculated as described in Materials and Methods. Resonances that disappeared upon binding are shown as negative bars; (**C**) Binding site mapping of full-length p53TAD, p53TAD1, and p53TAD2 peptides on the structure of MED25 ACID. Front (left) and rear views (right) are shown. The residues showing the chemical shift changes of ΔCS > 0.05 ppm are colored in yellow, and the disappeared residues are colored in red.

To determine the p53TAD-binding surface on the MED25 ACID, we carried out NMR experiments with ^15^N-labeled MED25 ACID. The addition of unlabeled full-length p53TAD into ^15^N-labeled MED25 ACID resulted in significant line broadening and chemical shift changes (Figure 2A), indicating that the complex underwent the intermediate to slow exchange on the NMR chemical shift time scale. The backbone resonance assignment of ^15^N-labeled MED25 ACID was performed based on the previously reported NMR assignment (BMRB ID: 17323) [11,12], and the NMR chemical shift perturbations (CSPs) were plotted against each residue of MED25 ACID (Figure 2B). Interestingly, mapping of the MED25 ACID residues, disappeared and significantly perturbed by NMR titration with p53TAD, revealed the two distinct binding surfaces on the opposite faces of MED25 ACID (front and back sides, Figure 2C). The p53TAD-binding sites were formed by β-strands (β4 and β7) and α1-helix in one face and β-strands (β1, β3, and β5) and α3-helix in the opposite face of MED25 ACID (Figure 2C). This binding site for p53TAD on MED25 ACID is the same as the previously identified VP16 TAD-binding sites [11,12].

The p53TAD contains two homologous ΦXXΦΦ sequence motifs for protein-protein interaction, referred to as p53TAD1 and p53TAD2. To test which motif was mainly responsible for interaction with MED25 ACID, we further performed NMR titration experiments with isolated p53TAD1 or p53TAD2 peptides (Figure 2). As shown in Figure 2A,B, each addition of unlabeled p53TAD1 or p53TAD2 peptides into ^15^N-labeled MED25 ACID caused the crosspeaks of MED25 ACID to significantly move. Although the full-length p53TAD contacted on the two distinct sites on opposite faces of MED25 ACID, the binding sites of p53TAD1 and p53TAD2 peptides were located on a hydrophobic groove in one face of MED25 ACID structure composed of β-strands (β4 and β7) and α1-helix (Figure 2C). The binding site for p53TAD2 peptide on MED25 ACID overlaps with that for H2 subdomain of VP16 TAD [11,12]. Noticeably, there was an obvious difference among them in the degree of CSPs caused by binding, with full-length p53TAD > p53TAD2 > p53TAD1.

**Figure 3:**
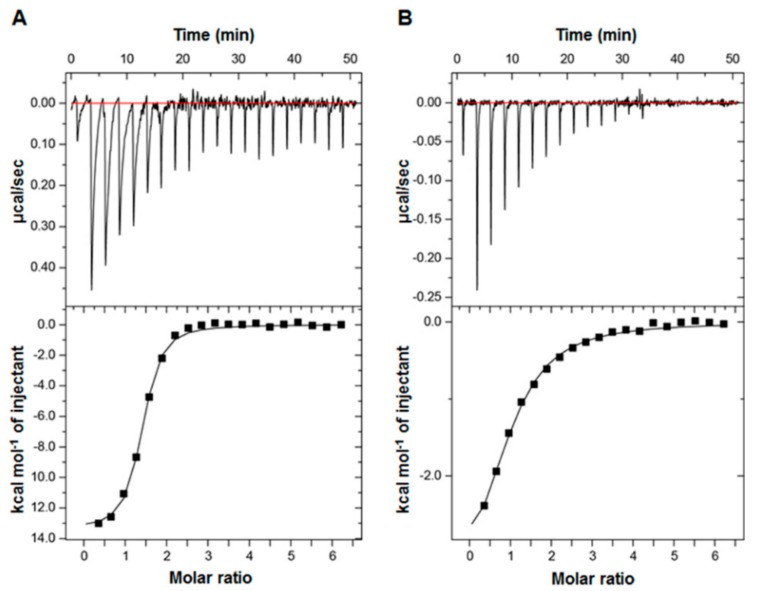
Binding affinity measurements of MED25 ACID with p53 p53TAD. The equilibrium dissociation constants (K_d_) between MED25 ACID and full-length p53TAD (K_d_, 0.8 ± 0.1 μM) (**A**) and p53TAD2 peptide (K_d_, 8.1 ± 0.8 μM); (**B**) were determined by isothermal titration calorimetry (ITC).

The difference in NMR CSP levels indicates the differential MED25 ACID-binding affinities among p53TAD, p53TAD1, and p53TAD2. To measure the binding affinity between them, we performed ITC experiments and determined the equilibrium dissociation constant (K_d_) between MED25 ACID and the p53TADs. The full-length p53TAD (residues 1–73) bound MED25 ACID with a K_d_ of 0.80 µM (Figure 3A). The p53TAD2 peptide bound MED25 ACID with a K_d_ of 8.10 µM (Figure 3B), although the K_d_ between the p53TAD1 peptide and MED25 ACID could not be determined by ITC. These K_d_ values were consistent with the binding affinities deduced from the CSP levels and chemical exchange regime from the NMR titration experiments (Figure 1 and Figure 2). The higher MED25 ACID-binding affinity of full-length p53TAD than those of p53TAD1 and p53TAD2 peptides may arise from cooperative binding mechanism of p53TAD1 and p53TAD2 motifs to MED25 ACID.

### 2.3. Structural Basis for the Interaction between MED25 ACID and p53TAD2 Peptide

**Figure 4:**
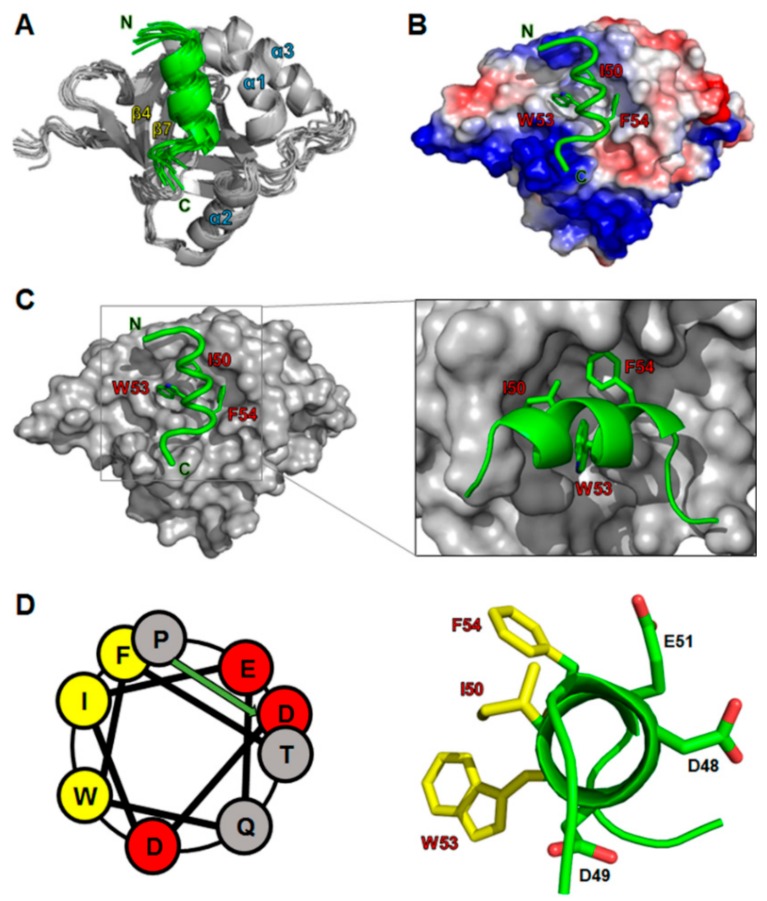
A structural model of the MED25 ACID/p53TAD2 peptide complex. (**A**) An ensemble of eight structural models are shown. MED25 ACID and p53TAD2 peptide are shown in gray and green, respectively; (**B**) Positive and negative electrostatic potentials on the molecular surface of MED25 ACID are colored in blue and red, respectively; (**C**) Molecular surface representation of the MED25 ACID (gray) complexed with p53TAD2 peptide (green); (**D**) The helical wheel diagram of p53TAD2 peptide.

Our NMR and ITC analyses showed that the p53TAD2 peptide was mainly responsible for binding MED25 ACID (Figure 2 and Figure 3). Based on the chemical shift perturbation data, we calculated a refined structural model for the complex between MED25 ACID and p53TAD2 peptide by using the HADDOCK 2.0 program (Figure 4). Ambiguous interaction restraints (AIRs) for the MED25 ACID and p53TAD2 peptide were defined on the basis of the NMR chemical shift perturbation data. In the complex structure, an amphipathic α-helix of the p53TAD2 peptide fitted into a hydrophobic cleft formed by the central β-barrel and α1-helix of MED25 ACID (Figure 4A). Within this binding pocket, the hydrophobic face of p53TAD2 α-helix contacted with the hydrophobic side chains of Leu458, Phe465, Met470, Leu514, Met523, and Leu525 in MED25 ACID. Noticeably, the highly conserved hydrophobic residues Ile50, Trp53, and Phe54 in p53TAD2 peptide were aligned into one face of the amphipathic α-helix, which was located at the binding interface with MED25 ACID, indicating that the hydrophobic interaction was a main driving force for the formation of the MED25 ACID/p53TAD2 peptide complex (Figure 4B,C). Furthermore, the complex model indicated that electrostatic interactions in p53TAD2 peptide contributed significantly to complex formation with MED25 ACID. The positively charged residues surrounding the p53TAD2-binding surface on MED25 ACID may serve as a complementary site for the electrostatic interaction with p53TAD2 peptide (Figure 4B). In particular, Arg466 in MED25 ACID, which is highly conserved among MED25 homologs, forms a salt bridge with Glu51 in p53TAD2 peptide as observed in the binding with the VP16 TAD H2 subdomain [11].

### 2.4. Mutational Analysis on Binding of p53TAD2 Peptide to MED25

**Figure 5:**
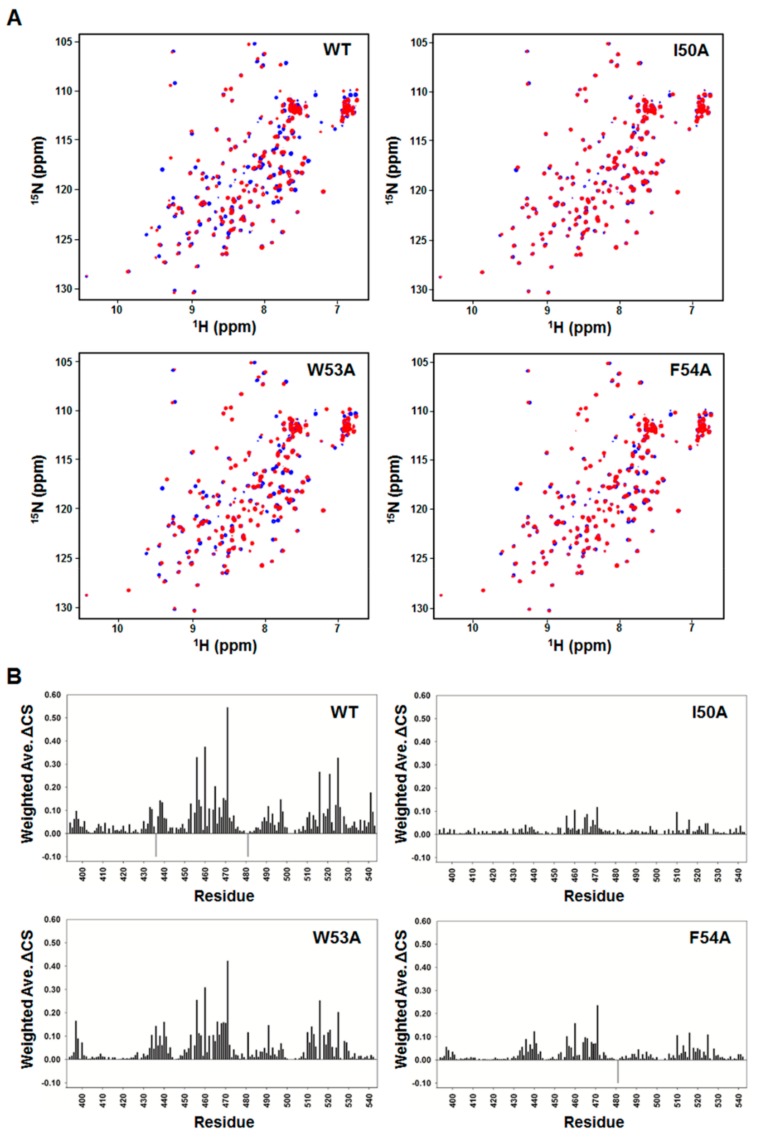
Mutational analysis on p53TAD binding to MED25 ACID; (**A**) Overlay of 2D ^1^H-^15^N HSQC spectra of ^15^N-labeled MED25 ACID in the absence (blue) or presence (red) of wild-type p53TAD2 peptide and mutant p53TAD2 peptides (I50A, W53A, and F54A) at a molar ratio of 1:1; (**B**) Chemical shift perturbations on MED25 ACID induced by binding to wild-type and mutant p53TAD2 peptides (I50A, W53A, and F54A) at a molar ratio of 1:1. Weighted ΔCS values were calculated as described in Materials and Methods. Resonances that disappeared upon binding are shown as negative gray bars.

To validate our structural model of the MED25 ACID/p53TAD2 peptide complex, we performed a mutational analysis on p53TAD2 peptide and NMR titration experiments with the mutant p53TAD2 peptides (Figure 5). Alanine mutations were introduced into the highly conserved Φ residues (Ile50, Trp53, and Phe54) in ΦXXΦΦ motif of p53TAD2 peptide. As shown in the 2D ^1^H-^15^N HSQC spectra of ^15^N-labeled MED25 ACID with mutant p53TAD2 peptide, the W53A mutation slightly diminished, and the I50A and F54A mutations nearly abolished the CSPs of MED25 ACID seen in the presence of the wild-type p53TAD2 peptide (Figure 5). These data indicated that the W53A mutation weakened, and the I50A and F54A mutations disrupted the binding of p53TAD2 peptide to MED25 ACID. Therefore, our results showed that the highly conserved hydrophobic residues are major binding determinants for MED25 ACID. In particular, Ile50 and Phe54 in the ΦΧΧΦΦ-binding motif of p53TAD make critical contributions to complex formation with MED25 ACID. This is consistent with the complex structure model, where the bulky hydrophobic side chains of Ile50 and Phe54 in p53TAD2 peptide fit into a hydrophobic groove in MED25 ACID (Figure 4C).

A previous mutagenesis study showed that ΦΧΧΦΦ motifs in p53TAD are important for transcriptional activity of p53 [34]. The mutational analyses with p53TAD (W53Q/F54S) and p53TAD (L22Q/W23S) showed that the residues Trp53 and Phe54 in p53TAD2 as well as Leu22 and Trp23 were critical for transcriptional activation in mammalian and yeast cells. It has been also shown that the p53TAD (W53Q/F54S) mutation that abrogates transactivation significantly impairs the binding of p53 to transcriptional coactivator p300 [35]. Taken together with these previous findings, our NMR observation that the ΦΧΧΦΦ motif (Ile50-Phe54) of p53TAD2 is involved in binding to MED25 ACID suggests that the interaction between p53TAD and MED25 ACID is well correlated with transcriptional activity of p53.

### 2.5. Conserved Binding Mechanism of MED25 with Intrinsically Unfolded Acidic TADs

According to amino acid composition of their TADs, transcriptional activators have been classified as acidic, Pro-rich, Gln-rich, and Ser/Thr-rich [36]. Acidic transcriptional activators such as p53, VP16, and ERM contain acidic TADs. Despite the intrinsically unfolded characteristics, the sequence alignment of the acidic TADs showed positionally conserved ΦXXΦΦ motifs for protein-protein interaction (Figure 1A). In this study, our results revealed a structural basis for the interaction of p53TAD with MED25. Noticeably, there was a close mimicry in the overall binding mode of MED25 among intrinsically unfolded, acidic p53TAD, VP16 TAD, and ERM TAD. First, although largely disordered in their free state, p53TAD2, VP16 TAD, and ERM TAD (residues 50–61) are induced to adopt amphipathic α-helices upon binding MED25 ACID [24,37]. Second, there is a high conservation in the major binding determinants in the ΦXXΦΦ motif. Similar to Phe442 in VP16 TAD [12] and Trp57 in ERM TAD [19], the residues Ile50 and Phe54 in the p53TAD2 peptide, aligned in one face of the amphipathic α-helix, point into the elongated hydrophobic groove of MED25 ACID and form key hydrophobic inter-molecular interactions. Lastly, our NMR data suggested that p53TAD contacts the distinct sites on opposite faces of MED25 ACID. This is consistent with the previous finding that H1 and H2 subdomains of VP16 TAD bind two distinct binding patches on opposite faces of MED25 ACID [11,12]. Thus, we suggest a mechanism of dual-site recognition of MED25 by intrinsically unfolded p53TAD, where the high-affinity binding of p53TAD2 to one face of MED25 ACID is followed by the low-affinity binding of p53TAD1 to the opposite face, resulting in a synergistic interaction in the p53TAD clamping the MED25 ACID.

## 3. Materials and Methods

### 3.1. Plasmid Construction and Peptide Synthesis

The human p53TAD (residues 1–73) and human MED25 ACID (residues 394–543) were cloned into the pGEX-4T vector (GE-healthcare, Chicago, IL, USA) containing an N-terminal glutathione-S-transferase (GST) tag and pET-21b vector (Novagen, Madison, WI, USA) containing a C-terminal His6 tag, respectively. The p53TAD1 (residues 15–29) and the wild-type and mutant p53TAD2 (residues 39–57) peptides were chemically synthesized and purified by Peptron Inc. (Daejeon, Korea).

### 3.2. Protein Expression and Purification

The expression vectors were transformed into the Escherichia coli BL21(DE3)RIL cells. Cells were grown to an optical density at 600 nm (OD600) of ~0.6 at 37 °C, and expression of the GST-fused p53TAD (residues 1–73) was induced at 18 °C with 0.5 mM isopropyl-β-d-thiogalactopyranoside (IPTG) for 16 h. Harvested cells were suspended in phosphate-buffer saline (PBS) (10 mM Na_2_HPO_4_, 140 mM NaCl, and 1.8 mM KH_2_PO_4_, pH 7.5) and lysed by sonication. After cell debris was removed by centrifugation, the supernatant was loaded onto a GST column (GE healthcare). After washing with PBS, the protein was eluted by elution buffer (50 mM Tris-HCl, pH 8.0, and 15 mM glutathione) prepared freshly. After cleaved with thrombin and dialyzed against Q-buffer (20 mM sodium phosphate, pH 6.4, 50 mM NaCl, and 2 mM dithiothreitol (DTT)), the protein was loaded onto a HiTrap-Q HP column (GE-Healthcare) and eluted by a 50–1000 mM linear gradient of NaCl. Expression of the His6-fused MED25 ACID (residues 394–543) was induced at 18 °C with 0.5 mM IPTG for 16 h. Harvested cells were suspended in 20 mM sodium phosphate, pH 6.5, 150 mM NaCl, and 10 mM β-mercaptoethanol and lysed by sonication. After cell debris was removed by centrifugation, the supernatant was loaded onto a Ni-NTA column (Qiagen, Hilden, Germany). After washing with 20 mM sodium phosphate, pH 6.5, 1 M NaCl, and 10 mM β-mercaptoethanol, and 20 mM sodium phosphate, pH 6.5, 150 mM NaCl, 10 mM imidazole, and 10 mM β-mercaptoethanol, the protein was eluted with 20 mM sodium phosphate, pH 6.5, 150 mM NaCl, 200 mM imidazole, and 10 mM β-mercaptoethanol. The protein was dialyzed against SP-buffer (20 mM sodium phosphate, pH 6.5, 50 mM NaCl, and 4 mM DTT), loaded onto a HiTrap-SP HP column (GE-Healthcare), and eluted by a 50–1000 mM linear gradient of NaCl. Finally, p53TAD and MED25-ACID were further purified by Superdex-75 gel filtration chromatography in 20 mM sodium phosphate, pH 6.5, 100 mM NaCl, 1 mM EDTA, and 4 mM DTT. The purity and homogeneity of the proteins were assessed using sodium dodecyl sulfate-polyacrylamide gel electrophoresis (SDS-PAGE). For NMR studies, uniform isotope labeling of proteins was obtained by growing the cells in M9 minimal medium supplemented with ^15^NH_4_Cl.

### 3.3. NMR Spectroscopy

All the NMR data were acquired using Bruker Avance II 800 and 900 spectrometers (Bruker, Billerica, MA, USA) equipped with a cryogenic probe at the Korea Basic Science Institute. The NMR samples were prepared in 20 mM sodium phosphate, pH 6.5, 100 mM NaCl, 1 mM ethylenediaminetetraacetic acid (EDTA), 4 mM DTT, and 10% (*v*/*v*) D_2_O. The 2D ^15^N-^1^H heteronuclear single-quantum correlation (HSQC) spectra of p53TAD were obtained in the absence or presence of the MED25 ACID at 25 °C. The 2D ^15^N-^1^H HSQC spectra of MED25 ACID were acquired in the absence or presence of the full-length p53TAD, p53TAD1, and wild-type and mutant p53TAD2 peptides at 25 °C. The 2D ^15^N-^1^H HSQC spectra were acquired with 4–8 scans with 1024 and 256 data points for ^1^H- and ^15^N-dimensions, respectively. The weighted ΔCS values for backbone ^1^H and ^15^N resonances were calculated by this equation: ΔCS = (Δ^1^H^2^ + (0.2 × Δ^15^N)^2^)^0.5^. All NMR data were processed and analyzed using nmrPipe/nmrDraw [38] and SPARKY 3 software (version 3, University of California, San Francisco, CA, USA, http://www.cgl.ucsf.edu/home/sparky/).

### 3.4. ITC Experiments

The equilibrium dissociation constants (K_d_) for MED25 ACID with full-length p53TAD (residues 1–73), p53TAD1 (residues 15–29), and p53TAD2 (residues 39–57) peptides were determined using an Auto-iTC200 Microcalorimeter (Malvern Panalytical, Malvern, UK) at the Korea Basic Science Institute. MED25 ACID of 600 µM was titrated into a solution of 20 µM p53TAD in a buffer containing 25 mM sodium phosphate, 100 mM NaCl, pH 7.0. Each p53TAD1 and p53TAD2 peptide of 600 µM was titrated into a solution of 20 µM MED25 ACID. Eighteen injections of 10 µL each were conducted at 25 °C, and the data were analyzed for one-site binding using MicroCal Origin^TM^ software (version MAN0577-02-EN-00, Malvern Panalytical, Malvern, UK).

### 3.5. Structure Calculation

The structure of the MED25 ACID/p53TAD2 peptide complex was calculated using the HADDOCK 2.0 program [39] in combination with crystallography and NMR system (CNS). Ambiguous interaction restraints (AIRs) were defined on the basis of the NMR chemical shift perturbation data. The active residues of MED25 ACID (Q455, M470, G491, K518, and L525) were defined as those showing chemical shift perturbations larger than the average (ΔCS > 0.1 ppm) or being substantially broadened at the protein to peptide ratio of 1:1, with relatively large per-residue solvent accessibility for either the side-chain or main-chain atoms. All of the surrounding surface residues near the active residues were defined as passive residues (Q456, L458, F465, V471, S516, and I521). Starting from the structures of MED25 ACID (PDB code: 2XNF) [11] and p53TAD2 peptide (residues 45–56) (PDB code: 2GS0) [40], rigid body energy minimization was performed, leading to 1000 rigid body docking solutions. In terms of intermolecular interaction energy, the 200 lowest structures were selected for rigid body-simulated annealing, followed by semi-flexible-simulated annealing in torsion angle space. Finally, the resulting structures were refined in explicit water by using simulated annealing in Cartesian space. The docking solutions were clustered based on positional root mean square deviation (r.m.s.d.) values by using a 3 Å cut-off. The complex models were selected for visualization based on their rmsd from the best energy structure and HADDOCK energy score. Figures of the structural models were drawn using the PyMOL software package [41].

## 4. Conclusions

Although interactions between transcriptional activators and the mediator coactivator complex are essential for eukaryotic transcription initiation, the structural basis for the interactions remains unclear. In this study, we characterized the molecular interaction between p53TAD and MED25 ACID using NMR spectroscopy. Taken together with ITC data, the NMR chemical shift perturbation showed that the p53TAD2 sequence motif was mainly responsible for the interaction with MED25 ACID, although full-length p53TAD engaged in contact on distinct sites of opposite faces of MED25 ACID. The refined structural model of MED25 ACID/p53TAD2 peptide complex revealed a mechanism of recognition, where an amphipathic α-helix of p53TAD2 peptide bound an elongated hydrophobic groove of MED25 ACID via hydrophobic residues Ile50 and Phe54. Our results suggest a highly conserved binding mechanism of intrinsically unfolded acidic TADs in the transcriptional activators p53, ERM, and VP16 for recruiting the mediator subunit MED25.
